# Removal Mechanism and Electrochemical Milling of (TiB+TiC)/TC4 Composites

**DOI:** 10.3390/ma15207046

**Published:** 2022-10-11

**Authors:** Shukai Fan, Xiaoyun Hu, Xin Ma, Yuting Lu, Hansong Li

**Affiliations:** National Key Laboratory of Science and Technology on Helicopter Transmission, Nanjing University of Aeronautics and Astronautics, Nanjing 210016, China

**Keywords:** TC4 composite material, electrochemical milling, rectangular cathode, removal mechanism

## Abstract

Titanium matrix composite (TiB+TiC)/TC4 has excellent physical properties and is a completely new composite material with great application prospects in the next generation of the aerospace field. However, there are problems, such as tool loss and material overheating, when using conventional processing methods. Electrochemical milling is a low-cost, high-efficiency processing method for difficult-to-machine metal materials with no tool wear. In this research, the feasibility of the electrochemical milling of (TiB+TiC)/TC4 and removal mechanisms during processing was reported for the first time. The feasibility of electrochemical milling is verified by the current efficiency experiment and basic processing experiment. Through the adjustment of the processing parameters, the final material removal rate increased by 52.5% compared to that obtained in the first processing, while the surface roughness decreased by 27.3%. The removal mechanism during processing was further performed based on the current efficiency experiment; three stages were observed and concluded during the electrolytic dissolution. This research proved that electrochemical milling is an excellent low-cost method for roughing and semi-finishing (TiB+TiC)/TC4 composites and provides guidance for better electrochemical milling in the titanium matrix composites.

## 1. Introduction

Titanium and titanium composites have extensive applications in the biomaterial [1], aerospace, and military fields owing to their excellent strength, high corrosion resistance [2], and outstanding heat resistance [3]. The titanium contents of F-22 and F-35 jets have reportedly reached 41% and 25% [4], respectively, and the titanium contents of Boeing passenger jets have also hugely increased from 0.5% to 14% [5]. The rapid development of aerospace technology demands superior materials for the weight reduction and structural optimization of aircraft engines [6] and fuselage parts [7]. Hence, the improvement in titanium and its composites has attracted increasing attention [8].

Continuous low-density, high-modulus reinforcement long fibers or uniformly distributed high-strength reinforcement particles (TiB [9], TiC [10], Graphene [11], SiC [12], and Al_2_O_3_ [13]) were added to the titanium alloy matrix to strengthen its wear resistance, heat resistance, elastic modulus, and other physical properties, generating titanium matrix composites (TMCs) [14]. Compared to titanium alloys, titanium composites used in aircraft engines can significantly increase the operating temperature, thereby increasing the engine thrust-to-weight ratio [15]. With the combination of the higher strength, plasticity, creep resistance, and mechanical properties of multiple reinforcement phases [16], TMCs undergo a noticeable weight reduction, have better high-temperature creep performance, can significantly simplify the structure of parts, and, thus, can become an essential material in the next generation of the aerospace field [17]. TiC and TiB are ideal reinforcing phases for titanium-based composites owing to their mechanical strength, high modulus, and low density [18].

Although the reinforcing particles substantially contribute to the enhancement of TMCs, processing TMCs is a big challenge because the material possesses both the high toughness of the titanium matrix material and the high hardness and brittleness of the reinforcing particles [19]. Machining them using conventional mechanical cutting methods [20,21] can cause severe tool wear [22], poor surface quality, and microcracks [23,24]. New unique processing methods such as Electrical Discharge Machining (EDM) create a heat-affected zone and remelting layers on their surface, which affect the surface quality [25,26]. Electrochemical Machining (ECM) can be used to machine the most difficult-to-machine metal conductive materials; it has not only a high MRR but also no tool wear, residual stress, recast layer, and microcracks; therefore, ECM is well suited for machining titanium matrix composite material [27].

The ECM process is closely related to the electrochemical dissolution characteristics of a material [28]. Wang reported the electrochemical dissolution behavior of Ti-48Al-2Cr-2Nb alloy in a low-concentration electrolyte and revealed the sample dissolution process in a 1% NaCl solution under different corrosion times [29]. Li revealed anisotropic behavior in different planes by studying the electrochemical anodic dissolution behavior of Ti-6Al-4V alloys produced by a laser solid–state forming additive manufacturing process [30]. Ge investigated the electrochemical dissolution behavior of cast superalloy K423A by evaluating the polarization curve and open circuit potential and proposed a qualitative model to explain the dissolution behavior of K423A in a NaNO solution [31].

Many studies have been conducted on the machining processes of Ti alloys and composites containing reinforcing phases. Feng explored the micro-mechanical properties and optimization of the mechanical-drilling process for TiBw/TC4 composites and established a failure mechanism for TiBw/TC4 composites [32]. Li studied the anisotropic electrochemical performance of LCD-produced TC4 alloy in a 15 wt.% NaCl solution, differences in electrochemical dissolution behavior, and electrochemical machinability in horizontal and vertical planes was investigated [33]. Wang investigated the material removal mechanism in the mechano-electrochemical milling of TC4 titanium alloy and established three models for its material removal process [34]. Liu explored the electrochemical machining of TB6 titanium alloy in a NaNO solution and successfully produced grooves and flat surfaces by electrochemical milling [35]. He optimized the tool cathode structure, created a more uniform flow field in TiB/7050 Aluminum Matrix Composite electrochemical milling, and reduced the flatness of the processing plane by 15.2% [36]. Ma evaluated the electrochemical turning method (ECT) for TMCs and verified the practical performance of the intermittent feed ECT method for machining titanium matrix composites [37]. Zhang investigated the electrochemical properties of (TiB+TiC)/TC4 composites, measured the polarization curves of (TiB+TiC)/TC4 in a 20% volume fraction NaCl solution, and analyzed the surface micro-topography after processing [38].

However, at this stage, there is a lack of (TiB+TiC)/TC4 composites’ in-depth analysis of the matrix material and the reinforcement phase particles’ behavior during the electrolytic dissolution process. In order to improve the processing efficiency and surface quality of this material in a wide range of applications, a prospective analysis of the material’s dissolution process behavior is essential. Therefore, this study investigated the electrochemical dissolution characteristics of the matrix of TC4 and reinforcements of 6.4% TiB and 1.6% TiC composites and explains the removal mechanism of the reinforcement phase based on its energy spectra and dissolution morphologies. Electrolytic milling is proved to be a promising low-cost roughing and semi-finishing method through feasibility experiments and mechanism analysis, which lays a foundation for the further popularization and application of this high-performance material in the aerospace field.

## 2. Materials and Methods

The titanium matrix composite material used in this study was provided by Zhejiang JiaTai Metal Technology Co., Ltd., reinforced by TiB and TiC particles, and the matrix material was Ti-6Al-4V (TC4) titanium alloy. The primary material components are shown in Table 1.

The volume fractions of the reinforcing phases TiB and TiC of the TMCs selected in this study were 6.4% and 1.6%, respectively. The physical properties of the (TiB+TiC)/TC4 composite are listed in Table 2.

### 2.1. Current Efficiency Curve Experiments

In ECM, owing to side reactions, the electricity passing through the anode is not entirely used for the dissolution of the anode metal [39]. The current efficiency, η, represents the ratio of the amount of electricity required to dissolve the anode metal material to the total amount of electricity passing through the circuit during machining [40]. Due to the difference between the mass of theoretical and actual material removal, it is necessary to plot the current efficiency curve of the (TiB+TiC)/TC4 composite material [41]. In the stable processing stage of ECM, the actual volume electrochemical equivalent must stay at a relatively stable value. Otherwise, the amount of anode material removed per unit time will fluctuate, and the processing gap cannot stabilize to a predetermined value [42]. 

During the ECM process, the electrochemical reaction of anode dissolution is complex, resulting in many types of oxidation products [43]. In general, an accurate volume electrochemical equivalent, ω, cannot be precisely obtained by calculation. Therefore, the product of the volume electrochemical equivalent, ω, and current efficiency, η, is selected to represent the actual volume electrochemical equivalent, as shown in Equation (1) [44]:

(1) $$\eta \omega =\frac{M_{1}-M_{2}}{\rho It}$$

where *ηω* represents the actual volume electrochemical equivalent, *M* is the initial mass of the anode material, *M* is the mass of the anode material after the test, *ρ* is the anode material density, *I* is the constant current, and *t* is the time for the current passing through the circuit [29].

This part of the experiment used the galvanostatic method [45]. The measured samples were (TiB+TiC)/TC4 composite cubes, having the same material, manufacturer, and processer. The sample pieces were cubes of 10 mm × 10 mm × 10 mm and were polished, ultrasonically cleaned, blow-dried, and weighed before the test. To improve the reliability of the measured current efficiency, a METTLER-TOLEDO ME204E electronic analytical balance with an accuracy of 0.0001 g was used, which satisfied the test requirements. A high-precision power supply (ITECH IT-6724, China) was selected to supply a constant current in test time t.

**Figure 1:**
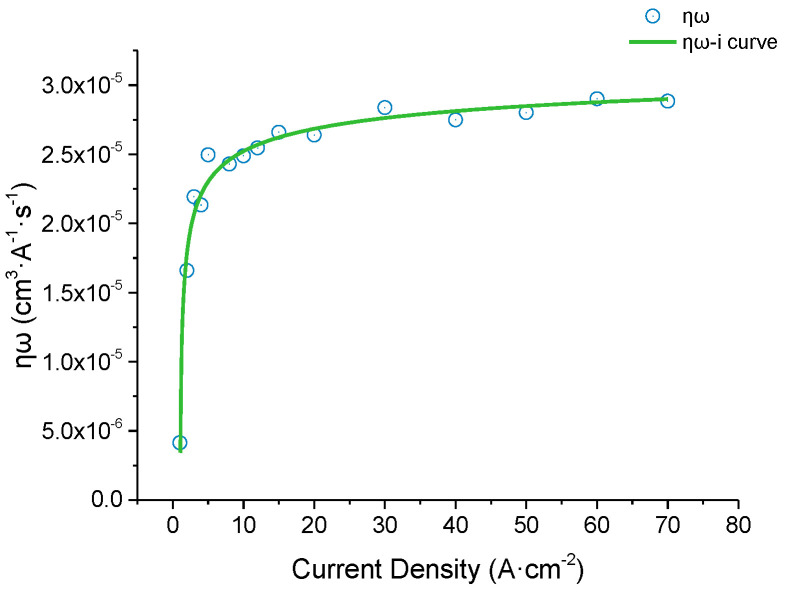
(TiB+TiC)/TC4 *ηω*-*i* curve, where *ηω* represents the actual volume electrochemical equivalent. As the current density increases, *ηω* first increases rapidly and then stabilizes.

The workpiece was then placed in an epoxy resin fixture. During the test, only the polished surface of the sample was corroded, and the rest of the surfaces were wrapped with a fixture to control the electrolytic corrosion area. After the experiment, the samples were ultrasonically cleaned, dried, and weighed. To ensure the reliability of the experimental data, each group of experiments was repeated thrice to reduce errors. NaCl solution is widely used in electrochemical machining of titanium alloy [33], and the machining efficiency increases with the rise of concentration [46]. At present, wt. 20% NaCl solution is commonly used as electrolyte [37,38]. Wt. 20% NaCl solution was used for subsequent experiments. The test parameter settings are listed in Table 3, and the measured efficiency curve (*ηω-i* curve) is shown in Figure 1.

### 2.2. ECM Experimental Setup

**Figure 2:**
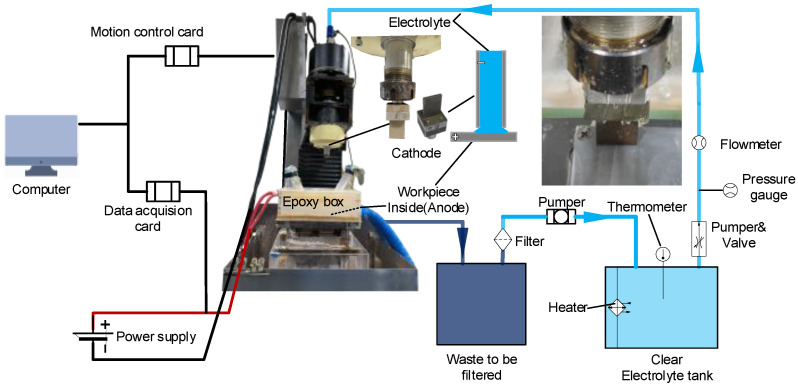
ECM system. The light blue line is the clear liquid transportation pipeline, and the dark blue line is the transportation pipeline of turbid liquid, which will be filtered after processing. Processing stage photo (Unpowered) is displayed in the upper right corner of the full image. The electrolyte flows from top to bottom inside the machine tool along the hollow electro-spindle, sprays from the opening of the tool cathode, and flows into the waste liquid tank from the blue pipe at the bottom right of the ECM machine.

The ECM system used in the electrolytic milling experiment is shown in Figure 2, including the computer program control and data acquisition, motion control, electrolyte circulation, and power supply.

The machining efficiency of a material is characterized by its MRR, which was calculated using Equation (2) [47], where Δ*m* is the mass difference before and after machining of the workpiece, measured using the same electronic analytical balance (METTLER-TOLEDO ME204E, Bonn Germany), and *t* is the machining time for a single milling pass.

(2) $$MRR=\frac{\Delta m}{\rho t}.$$

The quality of a machined surface is defined by its Ra. The roughness measurements were recorded using a roughness meter (Mahr GmbH∙Gottingen, Set M300-C, Göttingen, Germany), which averaged three independent measurements at different locations. The 3D contour shape of the machined workpiece was measured using a wide-area-three-dimensional measurement system (KEYENCE VR-5000, Osaka, Japan).

**Figure 3:**
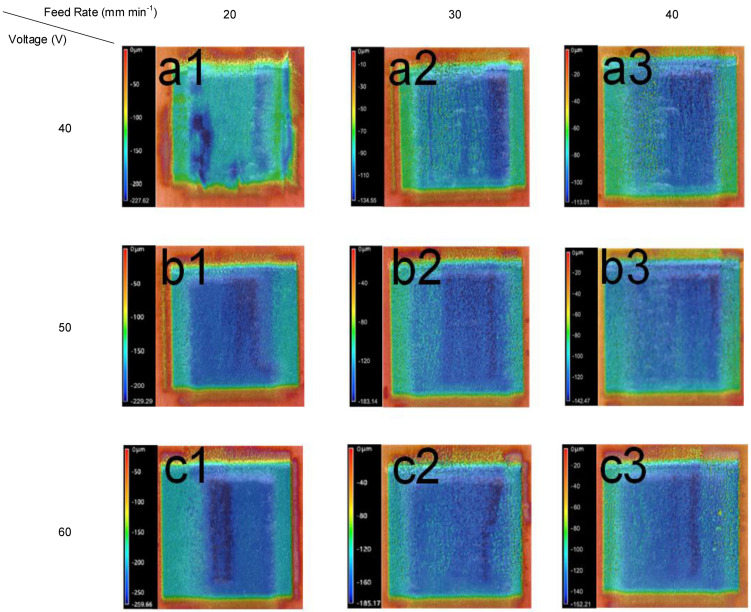
(TiB+TiC)/TC4 ECM surface 3D contour shape. The processing voltages of (**a1**–**a3**), (**b1**–**b3**), and (**c1**–**c3**) are constant at 40, 50, and 60 V, respectively. Regardless of (**a**)/(**b**)/(**c**), the feed rate is 20/30/40 mm min^-1^ from 1 to 3.

To validate the electrochemical milling feasibility for this material, experiments were next performed with a rectangular cathode under different machining parameters. The machining parameters are listed in Table 4 Parameters of the single-groove milling experiment. The macroscopic and microscopic shapes of the machined grooves are shown in Figure 3.

## 3. Results and Discussion

### 3.1. Effect of the Machining Voltage on the Experimental Results

**Figure 4:**
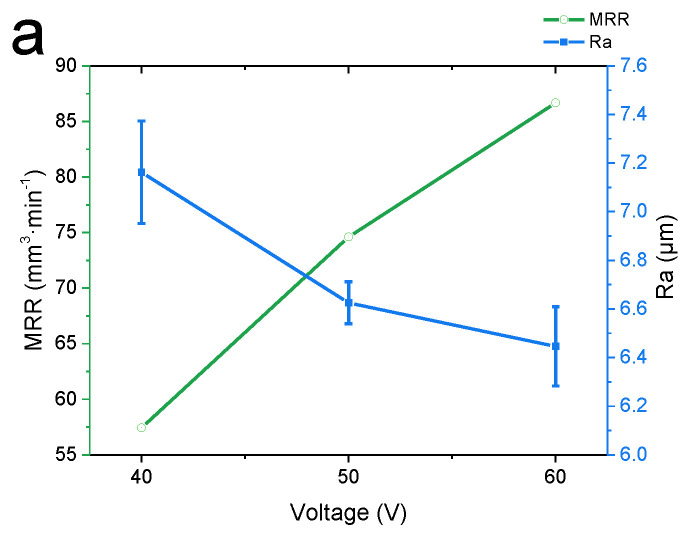

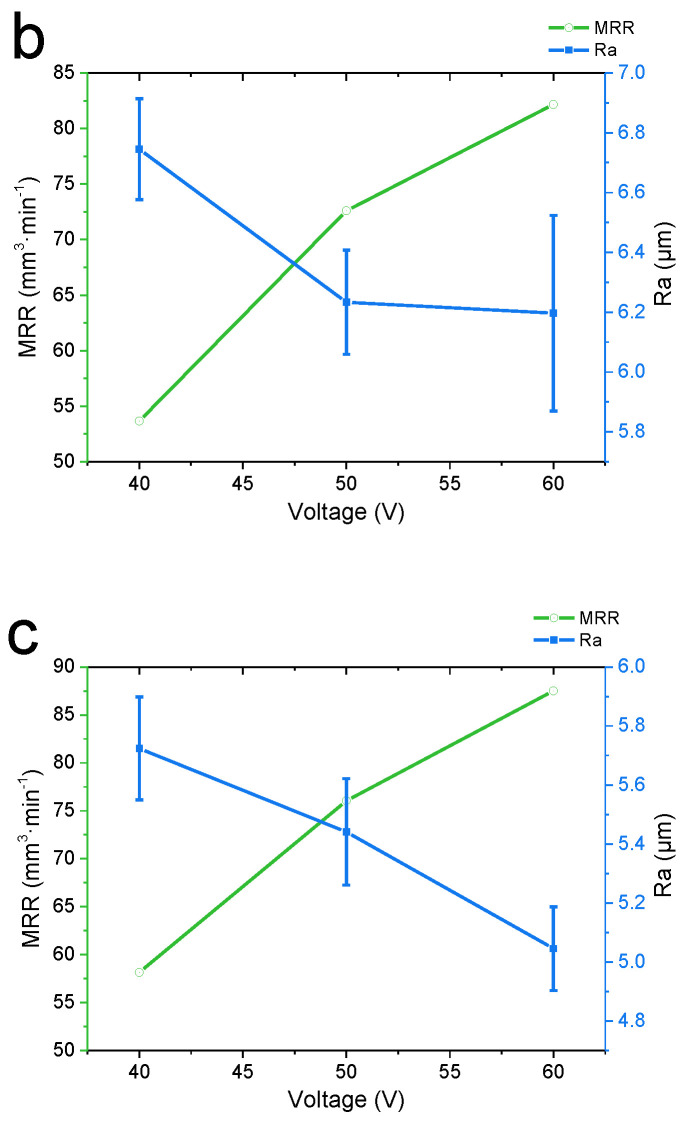
Material Removal Rate (MRR) and surface roughness (Ra) at different machining voltages. (**a**) Feed rate = 20 mm min^−1^, (**b**) feed rate = 30 mm min^−1^, and (**c**) feed rate = 40 mm min^−1^. Error bars represent the standard deviation of three individual measure results.

The machining voltage is an important factor affecting the ECM process. Figure 4 shows the changes in the machining efficiency and surface roughness of the workpiece with the increasing voltage.

As shown in Figure 4, as the voltage increased, the MRR showed a steady upward trend. At a feed rate of 40 mm·min^−1^ and an applied voltage of 60 V, the MRR reached a maximum value (87.51 mm^3^ min^−1^). Meanwhile, as the voltage increased, the Ra of the workpiece exhibited a steady downward trend. At 60 V, the Ra of the workpiece was approximately 75% of that at 40 V; an increase in the voltage increases the current and current density, and a high current density is conducive to the improvement of the surface quality. The Ra reached a minimum value of 5.045 μm at 60 V and 40 mm·min^-1^.

### 3.2. Effect of the Feed Rate on the Experimental Results

**Figure 5:**
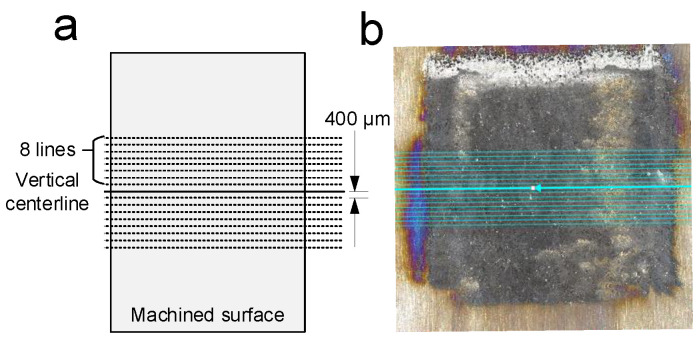
(**a**) Horizontal contour measuring method. The solid line at the center represents the center of the selected vertical direction, and the dashed lines on the upper and lower sides represent other measurements taken by averaging. (**b**) Physical image of measuring.

**Figure 6:**
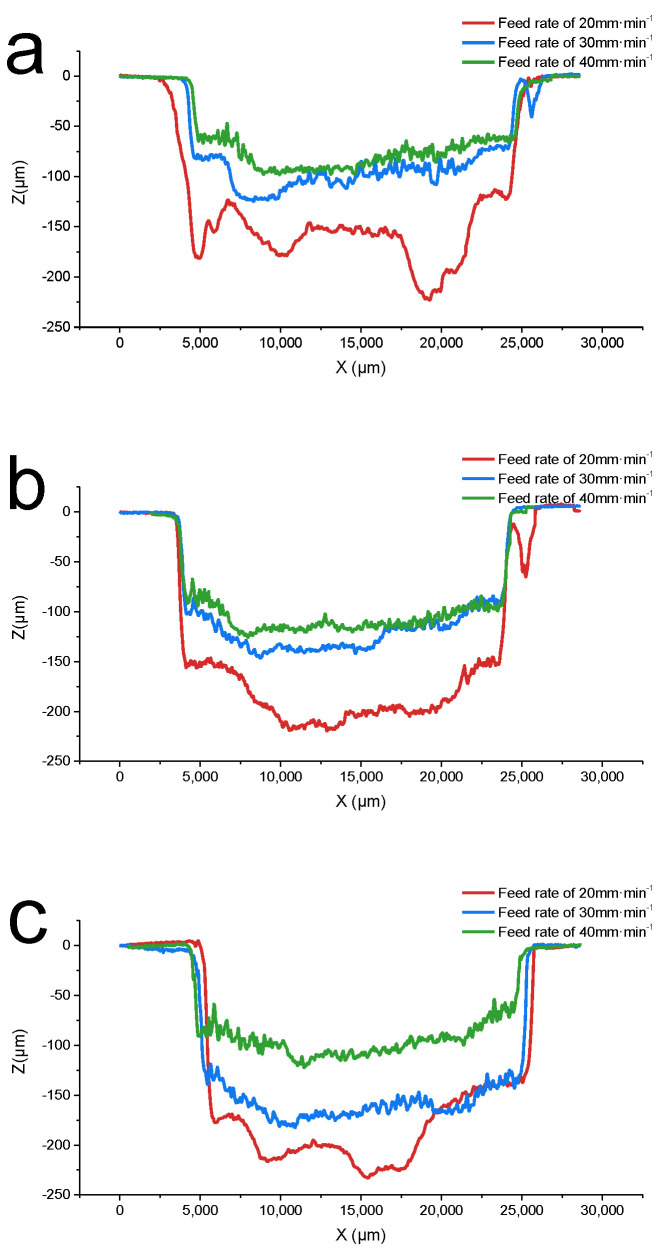
Horizontal profile at different feed rates. (**a**) Voltage = 40 V, (**b**) voltage = 50 V, and (**c**) voltage = 60 V. When feed rate = 20 mm·min^−1^ (red), the groove depth reaches the maximum, but the flatness of the groove is the worst. When feed rate = 40 mm·min^−1^ (green), the flatness is the best. The result of feed rate = 30 mm·min^−1^ (blue) is between 20 and 40, both for groove depth and flatness.

**Figure 7:**
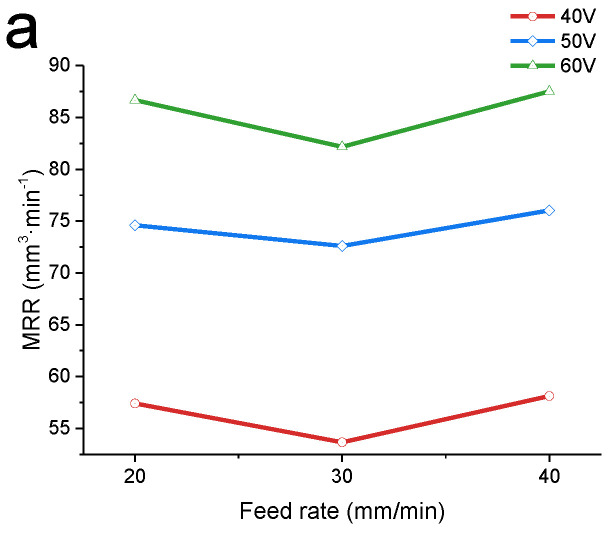

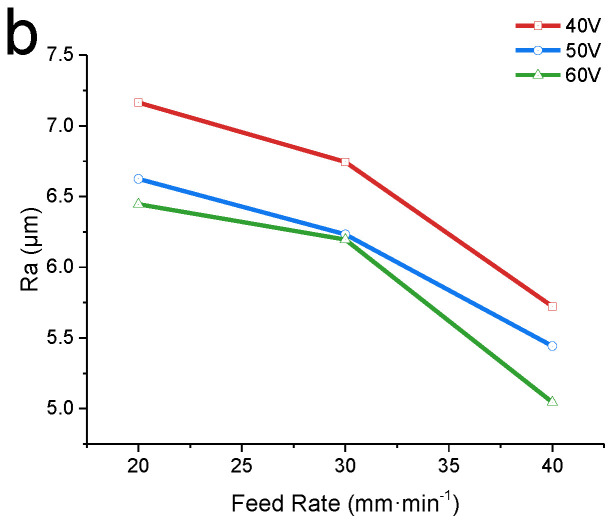
MRR and Ra at different machining voltages. (**a**) Different MRRs at applied voltage = 40 V (red), 50 V (blue), and 60 V (green). (**b**) Different Ra at applied voltage = 40 V (red), 50 V (blue), and 60 V (green). The effect of the feed speed on the MRR is not apparent, while surface roughness is significantly affected by the feed rate.

The following horizontal profiles were measured using the method shown in Figure 5. On the cross-section of the vertical center, eight horizontal lines with a distance of 400 µm from both sides were taken, and the horizontal contour measurement results of all the lines were averaged. Figure 6 shows the cross-section of the horizontal profile of a single-feed machined groove at different cathode feed rates:

Figure 7 shows the changes in the MRR and Ra under different parameters. The MRR was affected by the processing voltage; while increasing the processing voltage from 40 V to 60 V, the MRR increased by more than 50%. The Ra is significantly affected by the feed rate. Compared with mechanical turning, the MRR achieved by electrolytic machining is higher [48]. Therefore, a high voltage and feed rate of machining can effectively achieve high-efficiency and high-precision machining of the (TiB+TiC)/TC4 composite material.

### 3.3. Metallographic Analysis after ECM

**Figure 8:**
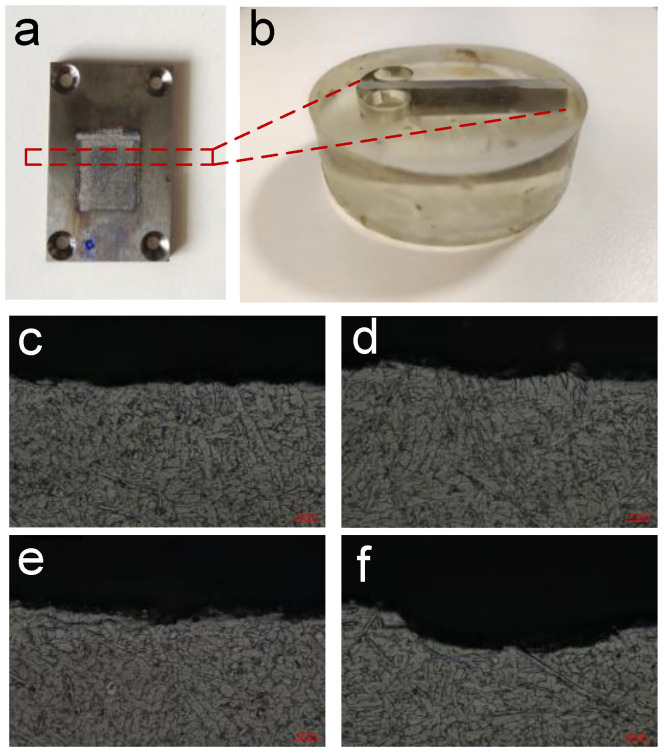
(**a**) Sample after single-groove milling experiment. The red dotted frame is cut by low-speed wire cutting, embedded in acrylic, (**b**) The re-polished sample. (**c**–**f**) Metallographic images after ECM processing.

All surfaces obtained under different machining parameters were inspected using a metallographic microscope (LEICA-DM4 M, Berlin, Germany), as shown in Figure 8. Multiple images were selected as the representative results of the metallographic analysis.

### 3.4. Experimental Discussion

Result of the current efficiency experiment: As shown in Figure 1, when the current density is lower than 10 A cm^−2^, the actual volumetric electrochemical equivalent increases sharply with the increasing current density. When the current density is greater than 10 A cm^−2^, the change in *ηω* tends to stabilize at approximately 2.7 × 10^−5^ (cm^3^·(A^−1^ s^−1^)). At low current densities (0–10 A cm^−2^), the actual volumetric electrochemical equivalent is significantly affected by the current density. During processing, if the current density is too low, the anodic etching speed is difficult to control, which quickly causes immense changes in the machining gap and processing stability [49]. Therefore, in actual machining, the current density should be greater than 10 A cm^−2^ to ensure the consistency of the machining gap and stabilize the processing quality. From the current efficiency experiment, it can be initially concluded that electrolytic milling can be used for the machining process of the material.

Result of the single-groove milling experiment: The macroscopic 3D contour of the machined surface obtained after the ECM processing is shown in Figure 3. With different combinations of parameters, significant differences can be seen in the shape and depth of the machined part. The machining depth increases with the voltage and decreases with the feed rate. The following is a detailed discussion of the influence of each parameter.

Effect of the machining voltage: Figure 7 shows how the MRR and surface roughness changed at different machining voltages. The current density has a great influence on the MRR and surface roughness of the workpiece. The surface quality generally increases with the increasing current density [50]. The current density increases with a higher voltage, so overall, the surface roughness of the workpiece decreases with the increasing voltage. A higher current means more electrons are exchanged per unit of time, and anode metal atoms lose electrons easier, so the material removal rate rises significantly with an increasing voltage. This is also mentioned by researchers in the electrochemical machining of other materials [36,51].

Effect of the feed rate: Figure 6 shows the cross-section of the horizontal profile of a single-feed machined groove at different cathode feed rates. The profiles were measured using the method in Figure 5. When the feed rate is increased, the cathode sweeps across the anode surface at a faster rate, reducing the time for the electrolytic reaction to occur, so the depth of the grooves decreased. However, since a larger area is swept in the same amount of time, longer grooves are created, which in turn increases the MRR [36]. The effect of the feed rate on the material removal rate is not as pronounced as that of the voltage, but its effect on the surface roughness is significant. With an increase in the feed rate, the Ra decreased from 7.165 to 5.045 μm. A faster feed rate means better surface uniformity; increasing it can be tried as one of the methods to further reduce surface roughness in the follow-up research.

Result of metallographic analysis: Under different machining parameters, the processed surface had no light-colored remelted layer separated from the main structure [52] and no microcracks extended inside. This indicates that the surface after electrolytic milling has no microscopic defects and exhibits good mechanical properties. Thus, electrolytic milling can effectively remove (TiB+TiC)/TC4, and the obtained surface is free of defects.

In conclusion, ECM is a promising method of processing (TiB+TiC)/TC4 composite material. However, the removal mechanism of the (TiB+TiC)/TC4 material remains unknown, which is essential to improve processing efficiency and surface quality. Therefore, based on previous experiments, the microscopic removal mechanism was studied and analyzed.

## 4. Removal Mechanism of (TiB+TiC)/TC4 Composites

**Figure 9:**
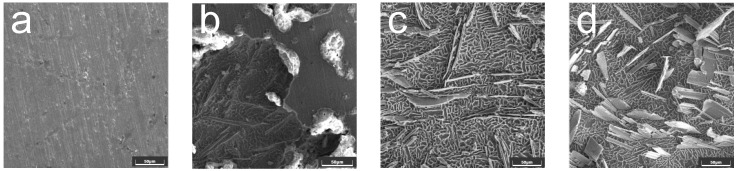
500× SEM images of samples at different processing times at current density = 30 A/cm^2^, 30 °C in a wt. 20% NaCl solution. (**a**) Processing time = 0 s, (**b**) processing time = 5 s, (**c**) processing time = 20 s, and (**d**) processing time = 100 s.

To further study the material removal mechanism, the surface morphologies of the samples at different processing times were investigated. The test was conducted at 30 °C in a wt. 20% NaCl solution. The galvanostatic method was used at a current density of 30 A/cm^2^. After processing at different times, the surface topography was inspected using an SEM (TESCAN, Vega 4, Brno, Czech Republic) and an energy-dispersive X-ray detector (EDX). The results are shown in Figure 9.

As shown in Figure 9b, in the initial stage of processing (0–5 s), the surface of the sample began to dissolve, and pits caused by cracks and electrolytic corrosion appeared; the reinforcing phase particles in the material were gradually exposed. After the processing duration reached 20 s, microscopic particles in the material were prominently displayed on the surface. In addition to the pits caused by electrolytic corrosion, concaves were also left by the peeling of the reinforcing phase material on the surface. Figure 9d shows the surface morphology after 100 s of processing. The reinforced phase particles were uniformly distributed on the matrix material, and similar to Figure 9c, no reinforced phase material subjected to electrolytic corrosion was observed, indicating that these microscopic particles did not participate in the electrolysis reaction. Their removal mechanism involves the corrosion of the matrix material TC4, complete dissolution of the surrounding base materials, and removal of microscopic particles by the high-speed flowing electrolyte.

**Figure 10:**
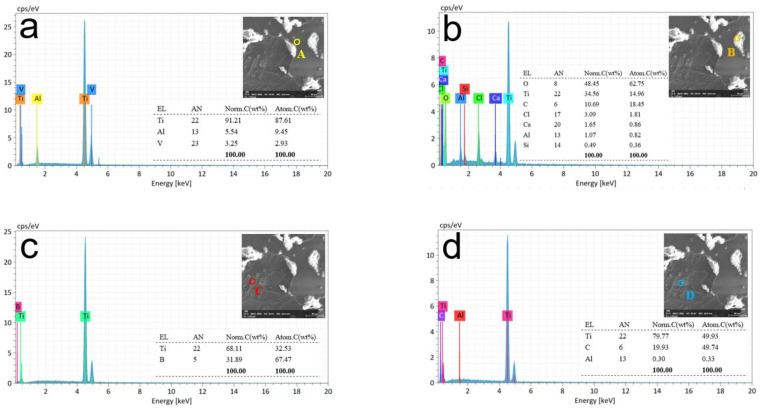
Energy spectrum analysis at different spots of processing time at 5 s. Different colored circles indicate measurement points at various locations. (**a**) Surface of the undissolved composite material. (**b**) Partially corroded surface material. (**c**,**d**) Reinforcing phase at the lower layer.

Figure 10 shows the surface morphology and energy spectrum analysis of the (TiB+TiC)/TC4 composite after the 5 s dissolving test, detected by SEM. EDX was used to measure four independent measurement points, as shown in Figure 9b, and the results are shown in Figure 10a–d.

As shown in Figure 10a, the microscopic morphology indicated that the surface of the composite material began to dissolve under the action of electrolysis. The figure shows the selected measurement point A in the yellow circle, which is the surface of the undissolved composite material. The elemental composition of the measurement results is Ti, Al, and V; therefore, the material at point A is matrix TC4 (Ti-6Al-4V). There is no oxygen element in the result, indicating that there is no presence of any oxidation product or oxide film at measurement point A. The absence of elements B and C at point A indicates that the distribution of reinforcing phase particles inside and on the surface of the material is not uniform. Figure 10b shows the selected measurement, point B (orange circle). The selected point was partially corroded but not completely dissolved. The elements in the measurement result included a large amount of oxygen, titanium, and some carbon and chlorine, whereas, at measurement point B (the white area), matrix TC4 was dissolved by electrolysis, leaving electrolytic impurities on the surface. The presence of element C indicates that the dissolution occurs first near the reinforcing phase, and we believe that the existence of this reinforcing phase can possibly reduce the corrosion resistance of the matrix. 

The red circle in Figure 10c represents measurement point C. The selected point was a long rod-like reinforcing phase part of the lower layer after the surface layer of the matrix was dissolved. The elemental composition in the measurement results was Ti and B; therefore, the material at point C was a TiB-reinforced phase material. The blue circle in Figure 10d is measurement point D, and the selected point is the ellipsoid-shaped reinforcing phase part of the lower layer after the surface layer of the matrix is dissolved. The elemental composition in the measurement results was Ti, C, and a minimal amount of Al. Therefore, the D point material was a TiC-reinforced phase material. Both C and D are completely dissolved regions. Rich TiB is detected in the C point, and the D point is rich in TiC, indicating that the dissolution first occurs near the reinforcement phase and the dissolution of pure matrix TC4 occurs later in the process.

**Figure 11:**
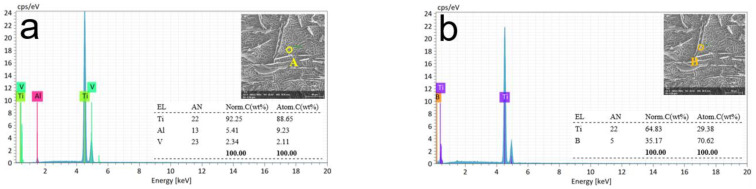

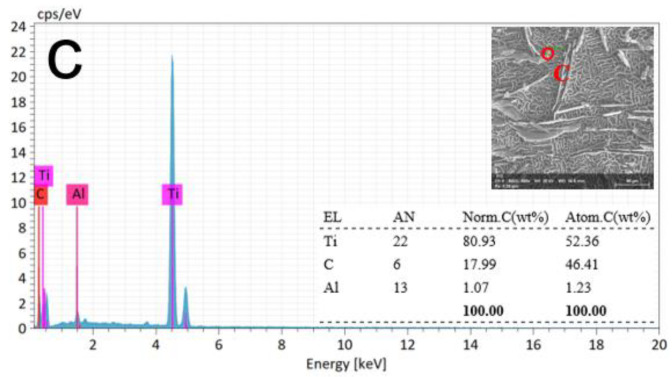
Energy spectrum analysis at different spots of processing time at 20 s. Different colored circles indicate measurement points at various locations. (**a**) Matrix material. (**b**,**c**) Reinforcing phase particles attached to the matrix.

Figure 11 shows the surface morphology and energy spectrum analysis of the (TiB+TiC)/TC4 composite after the 20-s dissolving test. The same SEM and EDX were used to measure three independent measurement points, as shown in Figure 9c, and the results are shown in Figure 11a–c. Compared with Figure 10, with the further progress of the dissolution, the top-surface matrix material was completely dissolved, and the inside matrix material/reinforcement phase began to appear. At this stage, the partially dissolved surface metal could not be found in the measurement result graph; therefore, we only assumed three measurement points and obtained three different measurement results.

In Figure 11a, point A had a honeycomb-like pattern inside the matrix material [38]. The elemental composition of the measurement results was Ti, Al, and V; therefore, the material at point A was matrix TC4 (Ti-6Al-4V). The orange circle in Figure 11b represents measurement point B. The selected point was a long, rod-like reinforcing phase. The elemental composition in the measurement results was Ti and B; therefore, the material at point C was a TiB-reinforced phase material. The red circle in Figure 11c represents measurement point C, which was a TiC-reinforced phase material. Compared with Figure 10, the entire surface layer has completely dissolved with time. The white area of the oxidation product is no longer observed. The reinforcing phase particles are clearly visible and inserted rather than deposited into the honeycomb-pattern matrix material TC4.

**Figure 12:**
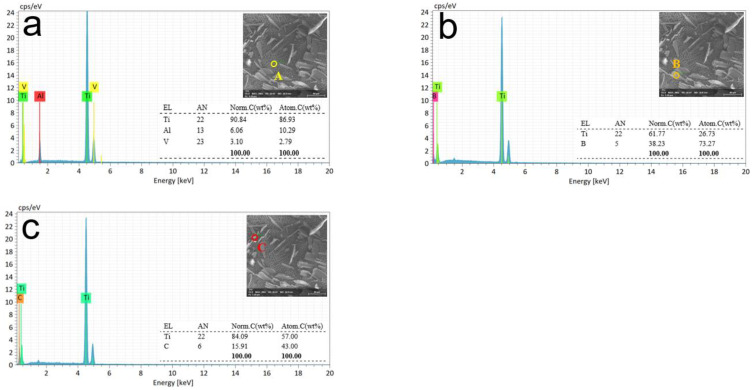
Energy spectrum analysis at different spots of processing time at 100 s. Different colored circles indicate measurement points at various locations. (**a**) Matrix material. (**b**,**c**) Reinforcing phase particles attached to the matrix.

Figure 12 shows the surface morphology and energy spectrum analysis of the (TiB+TiC)/TC4 composite after the 100 s current efficiency test. The same SEM and EDX were used to measure three independent measurement points, as shown in Figure 9d, and the results are shown in Figure 12a–c. Compared with Figure 11, as the dissolution process continues, the matrix material is dissolved layer-by-layer, and more reinforcement phase materials are exposed.

Similarly, the material at point A was matrix TC4 (Ti-6Al-4V). The material at point B was a TiB-reinforced phase material, and the C-point material was a TiC-reinforced phase material. Compared with the last stage, the reinforcement phase particles in the figure are further exposed from the matrix but remain attached to the matrix surface without being washed away by the high-speed flowing electrolyte. The reinforcement particles obviously have a great influence on the microstructure. Photographs of the micro-surface after machining demonstrate that a rough and semi-finish can be achieved with the non-contact electrochemical milling method, but higher quality surfaces may require a further finishing treatment.

From Figure 10, Figure 11 and Figure 12, the electrolytic dissolution process of the (TiB+TiC)/TC4 composite material can be described as follows: First, at 0–5 s, the surface layer of the TC4 metal material underwent an anodic dissolution reaction under the action of electrolysis, initially revealing the internal TC4 matrix material and reinforcing the phase particles; at 5–20 s, the TC4 metal material in the outermost layer completely dissolved, and different types of reinforcing phase particles were observed: a long rod-shaped (TiB ) and small ellipsoid (TiC). After 20 s, the dissolution entered the stable reaction stage, matrix material TC4 was continuously dissolved, and the number of exposed reinforcing phase particles increased. During the entire electrolytic reaction, as no electrolytic corrosion was observed on the surface of the reinforcing phase particles, only matrix metal TC4 was inferred to have participated in the electrolytic reaction [38].

**Figure 13:**
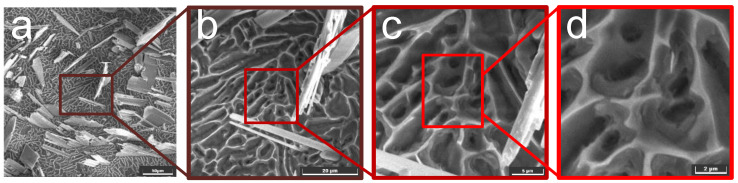
Higher-precision SEM images of 100 s dissolution processing time. (**a**) 500×, (**b**) 2000×, (**c**) 5000×, and (**d**) 10000×. The red box in each figure represents the zoomed-in part, resulting in the next figure.

Furthermore, high-precision SEM images of the samples were captured after the dissolution reaction for 100 s to study the removal mechanism of the reinforcing phase particles, as shown in Figure 13. The holes on the surface with increasing magnification are shown in Figure 13d. These holes were left behind when the reinforcing phase particles were dropped from the matrix. As the matrix material dissolved during electrolysis, the reinforcing phase particles that did not participate in the electrolytic reaction lost their surrounding support and were washed down from the surface of the substrate with the high-speed flowing electrolyte.

**Figure 14:**
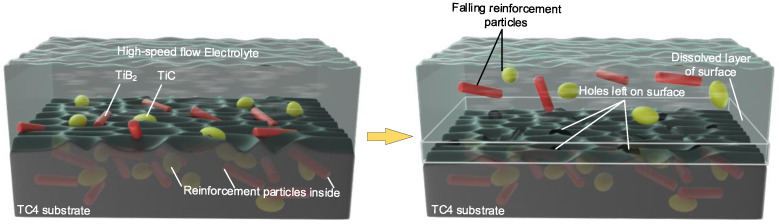
Electrochemical dissolution and removal mechanism of (TiB+TiC)/TC4 composite.

Figure 14 shows a schematic of the electrochemical dissolution and removal mechanism of the (TiB+TiC)/TC4 composite material. The reinforcing particles did not participate in the electrochemical dissolution reaction. As the electrolysis reaction proceeded, the matrix material TC4 was dissolved at a high speed, and the reinforcing phase particles were continuously exposed until the surrounding material was completely dissolved or insufficient to support the reinforcing phase particles. The reinforcing phase particles were continuously detached and left holes.

## 5. Conclusions and Outlook

For the titanium matrix composite with the matrix of TC4 and reinforcements of 6.4% TiB and 1.6% TiC, the relationship between the actual volume electrochemical equivalent and current density was analyzed, and a feasibility experiment was carried out to study the effect of the parameters on the processing. The removal mechanism of the (TiB+TiC)/TC4 composite during electrolysis was analyzed. The conclusions are as follows:

(1) As the current density increased from 1 A cm^−2^ to 70 A cm^−2^, the current efficiency curve showed a nonlinear trend, and the actual volume electrochemical equivalent first increased rapidly and then stabilized. So, there is a stable process for the electrolytic removal of this material at a current density higher than 10 A cm^-2^.
(2) In the voltage and feed speed ranges of 40–60 V and 20–40 mm·min^−1^, by adjusting the machining parameters, the material removal rate was increased by 52.5%, while the surface roughness was decreased by 27.3%. Under the processing parameters of 60 V (voltage) and 40 mm·min^-1^ (feed speed), the maximum MRR of 87.51 mm^3^ min^−1^ and the minimum surface roughness of 5.045μm were obtained, which met the rough and semi-finishing requirements.
(3) Three stages were observed and summarized in the process of electrolytic dissolution. At the beginning of the electrolysis reaction (0–5 s), the surface metal dissolved, exposing the underlying metal and reinforcing the phase materials. Subsequently, in a brief period (5–20 s), all of the top-surface metals were dissolved, but the reinforcing phase material did not participate in the electrolysis reaction. As the metal near the reinforcing phase particles dissolved, the reinforcing phase particles lost their support and then moved away by the high-speed flow electrolyte. Reinforcement particles have the possibility of reducing the corrosion resistance of the matrix TC4 material, and the reinforcement particles attached to the surface after processing also have a bad effect on the surface quality. From the mechanism and experiments, it is proved that electrolytic milling is an excellent low-cost method for roughing and semi-finishing (TiB+TiC)/TC4 composites.

In the follow-up research, co-relating the mechanism with the material removal rate and surface finish results is a promising direction for further research. Methods to improve processing efficiency can also be further explored.

## Figures and Tables

**Table 1:** TC4 titanium alloy base material composition.

Element	Ti	Al	V	Fe	C	N	H	O
wt.%	Balance	6.75	4.5	0.3	0.08	0.05	0.015	0.2

**Table 2:** Physical properties of (TiB+TiC)/TC4 composites at 20 °C.

Density (g/cm^3^)	Tensile Strength (MPa)	Yield Strength (MPa)	Elongations
4.5	>1020	>920	>6%
Elastic Modulus (GPa)	Thermal Conductivity (W mK^−1^)	Thermal Expansion Coefficient (°C^−1^)	Transformation Point (°C)
125	6.6	9.0 × 10^−6^	1035~1045

**Table 3:** Current efficiency measurement experiment parameters.

Experiment Parameters	Result
Electrolyte	Wt. 20% NaCl
Electrolyte Pressure (MPa)	0.2 ± 0.01
Electrolyte Temperature (°C)	30 ± 1
Machining gap (mm)	1
Electrolytic corrosion area (cm^2^)	0.25
Current Density (A cm^−2^)	1–70

**Table 4:** Parameters of the single-groove milling experiment.

Parameter	Value
Applied voltage (V)	40, 50, 60
Feed rate (mm min^−1^)	20, 30, 40
Electrolyte	20% NaCl
Electrolyte temperature (°C)	20
Electrolyte pressure (MPa)	0.2
Machining gap (mm)	0.3

## Data Availability

The data that support the findings of this study are available from the corresponding author, upon reasonable request.
